# Bioadhesive chitosan hydrogel with dynamic covalent bonds and sustained kartogenin release for endogenous cartilage regeneration

**DOI:** 10.3389/fbioe.2025.1606726

**Published:** 2025-07-29

**Authors:** Mingjing Li, Fan Li, Jian Xu, Li Zhu, Jiang Xiang, Chunquan Zhu, Zonghui Dai, Sen Tang, Fucheng Ouyang, Jiawen Yu, Xinwei Huang

**Affiliations:** ^1^ Department of Pediatric Orthopedics, Wuhan Fourth Hospital, Wuhan, China; ^2^ School of Bioengineering, Chongqing University, Chongqing, China

**Keywords:** catechol-modified chitosan, polyethylene glycol, bioadhesive dynamic hydrogel, kartogenin, endogenous cartilage regeneration

## Abstract

Articular cartilage defects are clinically prevalent yet lack effective therapeutic solutions. Recent advancements in acellular cartilage tissue engineering combined with microfracture techniques have shown promising outcomes. Injectable hydrogels have emerged as particularly attractive scaffolds due to their minimally invasive implantation and capacity to conform to irregular cartilage defects. However, their clinical application remains constrained by inadequate mechanical strength and insufficient bioadhesion. In this study, we developed a bioadhesive dynamic hydrogel by integrating catechol-functionalized chitosan with aldehyde-terminated four-arm polyethylene glycol (AF-PEG). When combined with KGN-loaded PLGA/PEG nanoparticles, this hydrogel system enables sustained KGN release while maintaining injectability, self-healing properties, and a 3D porous architecture. Mechanical characterization revealed superior bioadhesion strength (∼1,150 kPa) and compressive modulus (∼195 kPa). The hydrogel demonstrated excellent biocompatibility, significantly promoting bone marrow mesenchymal stem cells (BMSCs) proliferation, migration, and chondrogenic differentiation *in vitro*. *In vivo* evaluations showed superior ICRS and modified O’Driscoll histological scores in defects treated with the KGN-loaded chitosan hydrogels compared to controls. Histological analysis confirmed enriched type II collagen deposition in newly formed cartilage, exhibiting structural organization and integration with host cartilage comparable to natural tissue. This novel KGN-loaded bioadhesive dynamic hydrogel provides an optimized regenerative microenvironment for cartilage repair, demonstrating substantial translational potential for clinical applications.

## Introduction

Articular cartilage defects caused by trauma, infection, or osteoarthritis are very common and difficult to repair (Campos et al., 2019; Richter et al., 2016). Current surgical approaches for cartilage restoration primarily consist of osteochondral allografts, autologous chondrocyte implantation, osteochondral autografts, and bone marrow stimulation techniques such as microfracture and subchondral drilling (Gracitelli et al., 2016; Sommerfeldt et al., 2016). Among these interventions, microfracture-pioneered by Steadman in the late 1990s-has been regarded by some clinicians as the historical gold standard for treating focal cartilage defects, largely due to its arthroscopic feasibility and cost-effectiveness (Solheim et al., 2018). This technique involves creating perforations in the subchondral bone plate to facilitate the egress of bone marrow constituents, ultimately generating a fibrocartilaginous repair tissue within the defect. Numerous clinical studies have documented favorable short-term clinical outcomes following microfracture (Mithoefer et al., 2009). However, the newly formed fibrocartilage gradually degenerates over time, leading to suboptimal long-term outcomes. Additionally, the microfracture technique struggles to repair large scale cartilage defects. Therefore, by combining microfracture with cartilage tissue engineering techniques, it is possible to create a favorable microenvironment for the retention, proliferation, and chondrogenic differentiation of locally recruited stem cells (Case and Scopp, 2016; Pueyo et al., 2025). This approach enhances the secretion of cartilage extracellular matrix and holds promise for promoting endogenous hyaline cartilage regeneration.

Chitosan (CS), as a natural polysaccharide, exhibits excellent biocompatibility, making it widely applicable in cartilage tissue engineering (Comblain et al., 2017). Studies have demonstrated that chitosan-based hydrogels possess a three-dimensional structure analogous to the extracellular matrix (ECM) of cartilage, facilitating the diffusion and exchange of nutrients and metabolic products (Zhang et al., 2021a; Zhang et al., 2021b; Souza-Silva et al., 2024). The chitosan backbone contains abundant amino groups, which can dynamically bind with aldehyde groups under physiological conditions to form reversible imine bonds, enabling the preparation of dynamic hydrogels (Xu et al., 2025). The breakage and reformation of imine bonds endow hydrogels with injectability, viscoelastic adaptability, and self-healing properties. These characteristics mimic the biomechanical behavior of natural articular cartilage, thereby promoting the proliferation of chondrocytes and the secretion of cartilage matrix.

Among the numerous polymers containing aldehyde groups, polyethylene glycol (PEG) derivatives decorated by aldehyde groups have become commonly used crosslinking agents for preparing chitosan-based dynamic hydrogels due to their excellent biocompatibility (Andrade et al., 2022; Lin et al., 2024). Studies have reported that dialdehyde-functionalized PEG (DF-PEG), used as a crosslinker, forms dynamic hydrogels within 60 s when mixed with a chitosan solution, exhibiting notable self-healing properties (Wang et al., 2020). Mohrman et al. developed chitosan/DF-PEG hydrogels that promoted recovery in damaged central nervous systems (Mohrman et al., 2018). However, the suboptimal mechanical properties of these injectable hydrogels hinder their application in cartilage defect repair. Huang et al. (2023) reported an injectable methacrylated chitosan/PEG hydrogel, whose double-network system endows it with excellent mechanical strength, making it suitable for the treatment of intervertebral disc defects. Research indicates that polyaldehyde-functionalized PEG offers more crosslinking sites, resulting in hydrogels with superior gelation and mechanical properties compared to dialdehyde-modified PEG when combined with chitosan (Huang et al., 2016). Aldehyde-terminated four-arm PEG (AF-PEG) is a star-shaped PEG that is obtained by covalently bonding 4-formylbenzoic acid to the hydroxyl groups at each branch end through a carbodiimide coupling reaction. Due to its higher aldehyde group density compared to dialdehyde-functionalized linear PEG, AF-PEG was selected as the crosslinker in this study to prepare dynamic hydrogels with chitosan.

The injectability of dynamic hydrogels is particularly advantageous for repairing articular cartilage defects, as it allows minimally invasive implantation and precise filling of defects with varying sizes and geometries (Bertsch et al., 2023). However, it is well known that during clinical arthroscopic microfracture procedures, the intra-articular environment presents a highly dynamic aqueous milieu characterized by continuous irrigation with sterile saline and transient bleeding from cartilage defects. Traditional hydrogels struggle to achieve robust adhesion to surrounding cartilage under such wet conditions, leading to hydrogel detachment and compromised cartilage repair outcomes. Inspired by the exceptional underwater adhesion of mussels, studies have revealed that catechol groups can mediate interfacial adhesion in wet environments (Guo et al., 2020). Chitosan can be modified with catechol groups via carbodiimide reactions, yielding chitosan-based polymers that mimic the structure of mussel adhesive proteins, thereby exhibiting strong bioadhesive efficacy (Zhang et al., 2019). Zheng et al. (2020) utilized catechol-modified quaternized chitosan hydrogels to repair skin defects, demonstrating their excellent dermal adhesion, promotion of angiogenesis, and acceleration of wound healing. Furthermore, hydrogels containing catechol groups can immobilize and trap cells and signaling proteins from blood and bodily fluids after implantation into tissue defects (Souza Campelo et al., 2020). This enhances cellular adhesion and further accelerates local tissue regeneration. Therefore, this study aims to synthesize catechol-functionalized chitosan (CS-HCA) through carbodiimide-mediated conjugation with hydrocaffeic acid (HCA), followed by crosslinking with aldehyde-terminated four-arm polyethylene glycol (AF-PEG) to develop a novel dynamic hydrogel with enhanced bioadhesive properties. This strategy is expected to simultaneously improve the adhesive and mechanical characteristics of chitosan-based hydrogels for cartilage repair applications.

When the dynamic hydrogels adhere to the injured area, they can gradually release their carried bioactive factors to alleviate local inflammation, promote stem cell homing, and induce chondrogenic differentiation, thereby promoting endogenous cartilage repair. Commonly utilized bioactive factors include transforming growth factor-beta (TGF-β), insulin-like growth factor-1 (IGF-1), exosomes, and small-molecule drugs (Olov et al., 2022). Among these, Kartogenin (KGN), a non-protein small-molecule drug, induces chondrogenic differentiation of bone marrow mesenchymal stem cells (BMSCs) via the classical CBFβ-RUNX1 pathway (Cai et al., 2019). Studies demonstrate that KGN maintains its capacity to drive stem cell chondrogenesis even under inflammatory conditions. KGN can also maintain chondrocyte phenotype, alleviate joint inflammation, and reduce cartilage matrix degradation (Kwon et al., 2018). Furthermore, KGN exhibits exceptional stability, enabling room-temperature storage and delivery, and avoids receptor downregulation or desensitization issues associated with prolonged TGF-β use, highlighting its clinical potential. KGN has been encapsulated in nanoparticles due to its hydrophobicity. Researches also show that encapsulating KGN within poly (lactic acid-co-glycolic acid) (PLGA) or PLGA-PEG nanoparticles achieves sustained release, maintaining prolonged bioactivity to support continuous cartilage regeneration (Zhao et al., 2020; Almeida et al., 2020). Therefore, this study integrates KGN-loaded PLGA-PEG nanoparticles with CS-HCA hydrogel to develop a bioadhesive dynamic hydrogel capable of sustained KGN release. The hydrogel’s physical properties—including injectability, self-healing capability, microstructure, adhesive strength—and *in vitro* biocompatibility will be systematically evaluated. Subsequently, a rat knee joint cartilage defect model will be established to assess the hydrogel’s *in vivo* cartilage repair efficacy.

## Materials and methods

### Materials and reagents

Chitosan (deacetylation degree >95%, Jinan Hedebio Marine Biotechnology Co., Ltd., China); hydrocaffeic acid (HCA, China National Pharmaceutical Group Chemical Reagent Co., Ltd., China); 1-(3-Dimethylaminopropyl)-3-ethylcarbodiimide (EDC, Acros Organics, USA); N-hydroxysuccinimide (NHS, Acros Organics, USA); aldehyde-terminated four-arm polyethylene glycol (AF-PEG, Beijing J&K Scientific Technology Co., Ltd., China); Kartogenin (analytical grade, Wuxi Jiehua Pharmaceutical Technology Co., Ltd., China); PEG (China National Pharmaceutical Group Chemical Reagent Co., Ltd., China); PLGA (China National Pharmaceutical Group Chemical Reagent Co., Ltd., China). Other chemicals and reagents are listed in the Supplementary Table S1.

### Preparation and characterization of KGN-loaded PLGA-PEG nanoparticles (PLGA-PEG@KGN)

**FIGURE 1:**
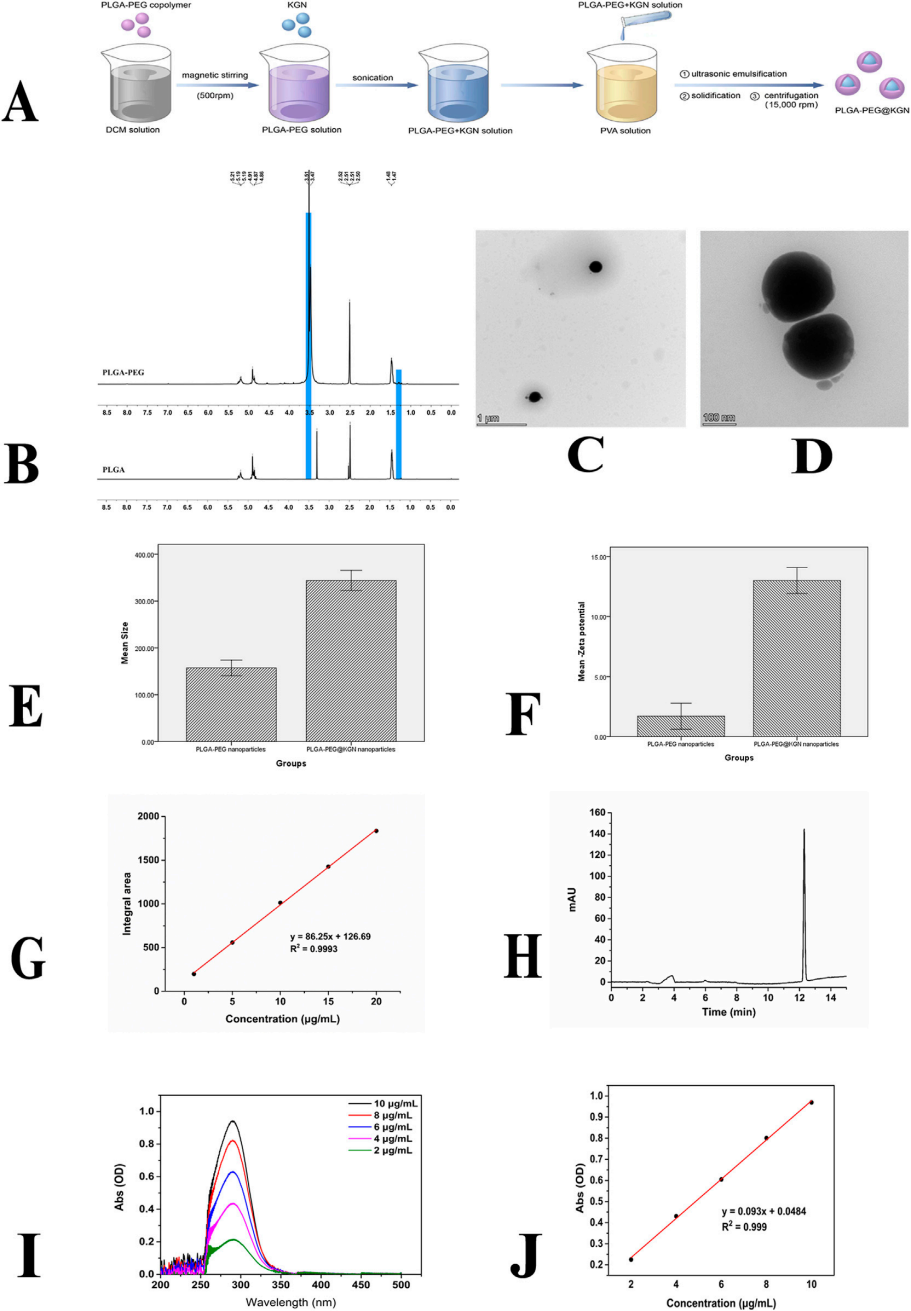
Characterization of PLGA-PEG nanoparticles. **(A)** Schematic diagram of nanoparticle preparation process. **(B)** ^1^H NMR spectra of PLGA and PLGA-PEG. **(C,D)** TEM images of PLGA-PEG nanoparticles. **(E)** Size distribution of PLGA-PEG and PLGA-PEG@KGN nanoparticles. **(F)** Surface Zeta potential of PLGA-PEG and PLGA-PEG@KGN nanoparticles. **(G,H)** Standard curve **(G)** and chromatogram **(H)** of PLGA-PEG@KGN nanoparticles analyzed by high-performance liquid chromatography (HPLC). **(I,J)** UV spectrophotometer analysis of nanoparticle encapsulation efficiency and drug loading capacity. Full-wavelength scan **(I)** and standard curve **(J)** of KGN.

The PLGA–PEG copolymer and PLGA-PEG nanoparticles were synthesized as previously reported (Almeida et al., 2020). The brief preparation procedure is illustrated in Figure 1A. 100 mg of PLGA-PEG was dissolved in 5 mL of dichloromethane (DCM) under magnetic stirring at 500 rpm until complete dissolution. Subsequently, 20 mg of KGN was added to the PLGA-PEG solution, followed by sonication for 5 min to ensure homogeneous drug dispersion. The KGN-loaded PLGA-PEG solution was slowly dripped into a polyvinyl alcohol (PVA) solution (1:5, v/v) for primary emulsification under ultrasonication, with continuous cooling in an ice bath. The solvent was rapidly removed using a rotary evaporator (40°C, 100 rpm, and a vacuum pressure of −0.08 MPa) until the emulsion transitioned from turbid to semi-transparent, indicating complete nanoparticle solidification. The emulsion was then transferred to centrifuge tubes and centrifuged at 15,000 rpm for 30 min to pellet the nanoparticles, after which the supernatant was discarded. The precipitate was resuspended in deionized water, and the centrifugation process was repeated three times to thoroughly remove residual free PVA and unencapsulated KGN. The washed nanoparticles were freeze-dried for 24–48 h and finally stored in airtight, light-protected containers at −20°C.

Chemical structure of PLGA-PEG was examined by ^1^H nuclear magnetic resonance (^1^H NMR) spectroscopy. The size distribution of the nanoparticles was analyzed using dynamic light scattering (DLS). Zeta potential was measured via electrophoretic light scattering (ELS). Morphological characterization of the nanoparticles was conducted using transmission electron microscopy (TEM). Drug encapsulation efficiency and loading capacity were determined by ultraviolet-visible (UV-Vis) spectrophotometry and High Performance Liquid Chromatography (HPLC). Surface chemical properties of the nanoparticles were examined using Fourier-transform infrared (FT-IR) spectroscopy. The *in vitro* release behavior of KGN was evaluated through a dynamic dialysis method monitored by UV-Vis spectrophotometry.

### Preparation and characterization of catechol-functionalized chitosan polymer (CS-HCA)

**FIGURE 2:**
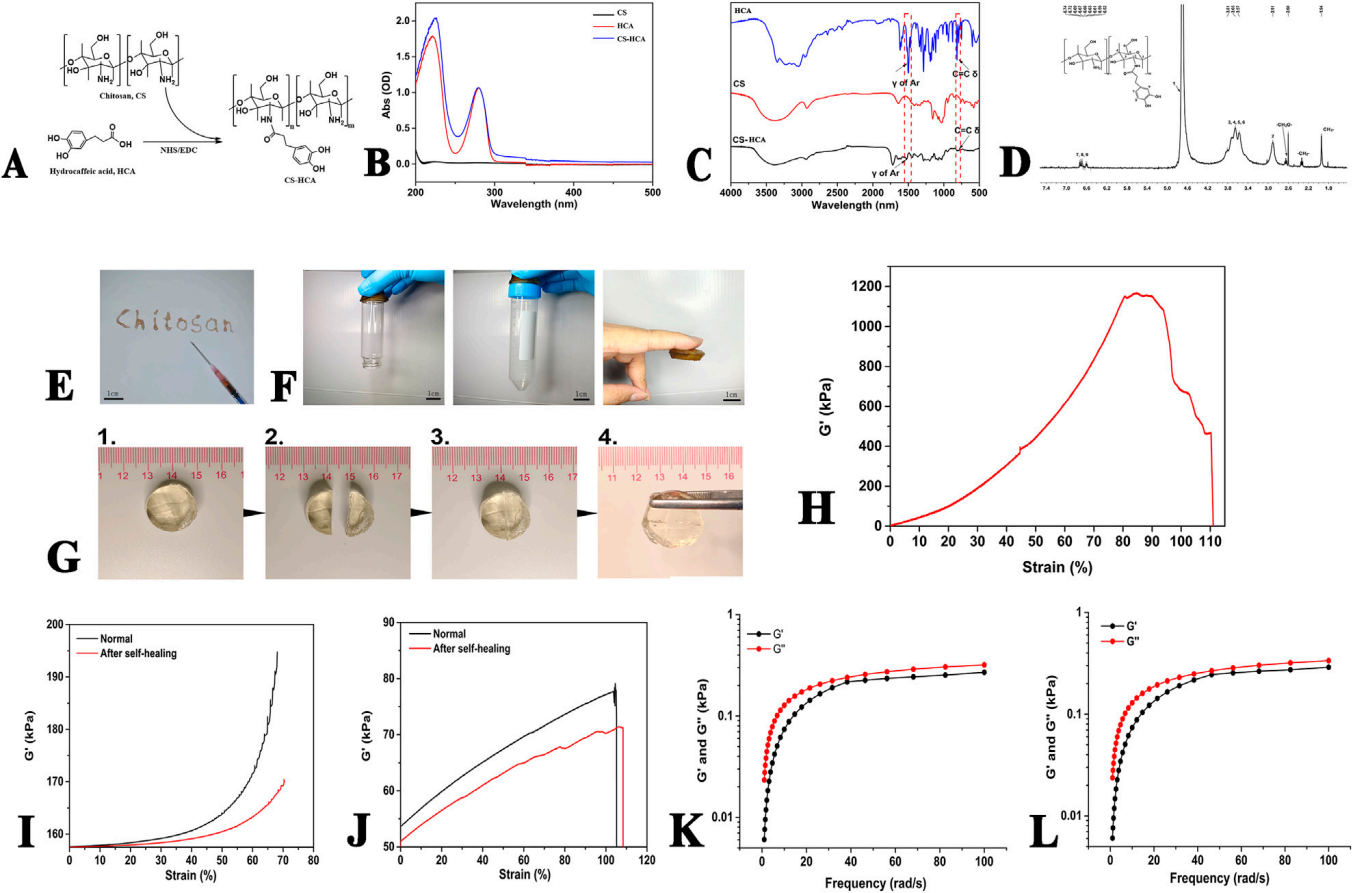
Characterization of CS-HCA hydrogels. **(A)** Synthesis route and chemical structure of CS-HCA polymer. **(B–D)** UV-Vis **(B)** FT-IR **(C)** and ^1^H NMR **(D)** spectra of CS-HCA polymer. **(E)** Image of the injectability of hydrogel at room temperature. **(F)** Images of the hydrogels adhesion to glass, plastic and skin surfaces. **(G)** Image of the self-healing process of hydrogel. **(H)** Adhesive strength of the hydrogels. **(I,J)** DMA results of the CS-HCA hydrogels. Compressive **(I)** and tensile **(J)** moduli of the hydrogels before fracture and after self-healing. **(K,L)** Rheological analysis of CS-HCA **(K)** and CS-HCA@PLGA-PEG@KGN **(L)** hydrogels (G', storage modulus, G'', loss modulus).

Figure 2A illustrates the grafting of catechol groups onto chitosan through an EDC/NHS-mediated conjugation reaction. (1) Dissolve 500 mg of chitosan (CS) in 45.5 mL of deionized water (pH ∼1.6) under continuous rapid stirring until a transparent light-yellow colloidal solution is formed. (2) Adjust the pH of the colloidal solution to 5.4 by dropwise addition of NaOH solution, resulting in a turbid colloid. Then, add 591 mg of hydrocaffeic acid (HCA) and continue stirring until the colloid becomes transparent again (pH ∼3.86). (3) Dissolve 1,244.8 mg of EDC (1-ethyl-3-(3-dimethylaminopropyl)carbodiimide) and 746.75 mg of NHS (N-hydroxysuccinimide) in 50 mL of an ethanol/deionized water mixture (1:1, v/v). Add this solution dropwise to the CS-HCA colloid. Adjust the pH to 4.6 with 1M HCl under vigorous stirring. (4) Dialyze the mixture using a regenerated cellulose dialysis membrane (MWCO: 12–14 kDa) against deionized water (pH 3.0–3.5) for 48 h, followed by dialysis against deionized water (pH 5.0) for 4 h. Freeze-dry the purified product to obtain the dried CS-HCA polymer. Store the final product under inert gas at −40°C.

Confirm the successful synthesis of CS-HCA via ^1^H NMR, FT-IR, and UV-Vis spectroscopy.

### Preparation of KGN-loaded CS-HCA bioadhesive dynamic hydrogel

(1) CS-HCA Solution Preparation: Weigh 500 mg of CS-HCA powder and dissolve it in deionized water under continuous stirring to prepare a 1.5% (w/v) homogeneous solution. Filter the solution to obtain a sterile formulation. (2) AF-PEG Solution Preparation: Weigh 500 mg of AF-PEG powder and dissolve it in deionized water. Filter the solution to ensure sterility. (3) Mix 5 mL of the CS-HCA solution with an equal volume of AF-PEG solution thoroughly. Transfer the mixture into a 12-well plate for molding, resulting in the formation of CS-HCA hydrogel. (4) Weigh 100 mg of PLGA-PEG@KGN nanoparticles, disperse them in deionized water, and homogenize via stirring. Add the nanoparticle suspension to the preformed hydrogel solution and mix rapidly until uniformity is achieved. Mold the final composite in a 12-well plate to obtain the KGN-loaded CS-HCA hydrogel (CS-HCA@PLGA-PEG@KGN). FT-IR spectroscopy analysis was performed to investigate the interactions between CS-HCA hydrogels and KGN nanoparticles.

### Rheological characterization of CS-HCA hydrogels

A 5 wt% CS-HCA hydrogel was prepared in PBS buffer (pH 7.4), and its viscoelastic properties were evaluated using an Anton Paar rheometer equipped with a PP50 stainless steel plate (50 mm diameter). The storage modulus (G′), representing the elastic component, and the loss modulus (G″), representing the viscous component, were measured to assess the hydrogel’s mechanical behavior. A hydrogel exhibits dominant elastic properties (G' > G″) in the gel state, while viscous behavior prevails (G'' > G′) in the sol state. The sol-gel transition point occurs when G' = G″, indicating a balance between viscous and elastic responses. For the frequency sweep test, the following parameters were applied: a gap distance of 0.5 mm, angular frequency range of 0.1–100 rad/s, constant strain of 1%, temperature of 37°C, and a normal force F_N_ of 0 N.

### Evaluation of hydrogel injectability, self-healing, and bioadhesive properties

(1) Prepare a 5 wt% CS-HCA hydrogel solution. Aspirate 0.5 mL of the sol into a 1 mL syringe and assess macroscopic injectability via extrusion-based writing tests. (2) Cut a cylindrical hydrogel into two-halves. Bring the fractured surfaces into contact and observe interfacial integration to evaluate macroscopic self-healing capability. (3) Apply the hydrogel to plastic, glass, and skin substrates to assess its adhesive performance under varying surface conditions.

### Mechanical properties measurement

The stress/strain sweep of CS-HCA hydrogels before fracture and after self-healing was performed using a dynamic mechanical analyzer (DMA). Alternating amplitude strains (large strain to disrupt the gel structure and small strain to observe structural recovery) were applied to evaluate the self-healing properties of the hydrogel. The testing procedure was as follows: the angular frequency was maintained at 10 rad/s, with alternating strains of 1% and 1,000%, the test temperature was set at 37°C, and the normal stress (F_N_) was 0 N.

### Characterization of hydrogel microporous structure

Prepare hydrogel solutions with solid contents of 1 wt%, 2 wt%, and 5 wt% in PBS (pH 7.4). Rapidly quench the gels in liquid nitrogen to embrittle them, then fracture the samples with tweezers. Freeze-dry the fractured gels, sputter-coat with gold, and examine cross-sectional pore structures using scanning electron microscopy (SEM).

### Determination of hydrogel porosity

Add anhydrous ethanol (V1) into a clean, dry graduated cylinder. Submerge a pre-weighed dry hydrogel scaffold in the ethanol, ensuring complete immersion. Apply negative pressure evacuation until the scaffold is fully saturated (no bubbles emerge), and record the ethanol volume (V2). Remove the scaffold and measure the residual ethanol volume (V3). Calculate porosity using the formula: $\text{Porosity }\left(\%\right)=\left(V1-V3\right)/\left(V2-V3\right)\times 100\%.$ All the experiments were repeated six times.

### Adhesion strength measurement

Porcine skin specimens (5.0 cm × 1.5 cm) were cut and fixed onto transparent glass slides (25.4 mm × 76.2 mm). Two porcine skin specimens were adhered using the hydrogel solution and maintained at room temperature for 1 h prior to testing. Specimens were stretched on a universal testing machine equipped with a CMT 100N force sensor at a crosshead speed of 5 mm/min under controlled conditions (26°C, 50% humidity) until separation occurred. Maximum load and displacement were recorded. Shear strength was calculated as the maximum load divided by the overlapping contact area. All experiments were performed in quintuplicate, and the mean values are reported.

### *In vitro* degradation

The initial mass of the hydrogel scaffold was recorded as W0. The hydrogel was placed into a centrifuge tube prefilled with an equal volume of PBS (pH 7.4). Complete submersion of the hydrogel in PBS was ensured, followed by incubation at 37°C for 1–4 weeks. At predetermined weekly intervals, the hydrogel scaffolds were retrieved, surface moisture was gently removed, and the remaining mass was recorded as W1. The degradation rate (%) was calculated using the formula:

$$\text{degradation ratio }\left(\%\right)=\left(W0\;-\;W1\right)/W0\times 100\%.$$

### Isolation and culture of bone marrow mesenchymal stem cells

All experimental protocols were approved by the Animal Experimental Ethics Committee of Chongqing Western Biomedical Technology corporation (No.20240415S0201231 [01]).

SD rats were used to extract BMSCs according to the method described in the literature (Maridas et al., 2018). The cells were seeded into culture flasks at a density of 1 × 10^6^ cells/mL and cultured in DMEM/F12 medium supplemented with 10% fetal bovine serum (FBS) and penicillin-streptomycin. Second-passage (P2) cells with good growth status were selected for subsequent experiments. The cells were divided into three groups: 2D conventional culture group (2D), CS/HCA hydrogel group (CS/HCA), KGN-loaded CS/HCA hydrogel group (CS-HCA@PLGA-PEG@KGN). The 2D group was cultured under standard 2D conditions, while the other two groups were cultured on CS/HCA or CS-HCA@PLGA-PEG@KGN hydrogels respectively.

### *In vitro* BMSCs proliferation

The proliferation of BMSCs in each group was evaluated using the CCK-8 assay at 24, 48, and 72 h post-culture. After termination of cultivation, cells were treated with trypsin, resuspended in culture medium, and seeded into 96-well plates. Each well received 100 μL of cell suspension supplemented with 10 μL of CCK-8 solution. Following 1–4 h of incubation, absorbance (A) at 450 nm was measured using a microplate reader. Six replicates of each group were studied.

### *In vitro* BMSCs migration

Cell migration ability was evaluated using the scratch assay and Boyden Transwell chamber assay (24-well plates with polycarbonate membranes, pore diameter 8.0 μm, membrane thickness 6.5 mm).

Scratch assay: BMSCs from each group were processed as planned, adjusted to a density of 2 × 10^5^ cells/mL, and seeded into 6-well plates with 2 mL of cell suspension per well. When cell confluence reached 80%–90%, the medium was aspirated. A sterile 10 μL pipette tip was used to create a linear scratch perpendicular to the plate surface. BMSCs were rinsed 3 times with PBS to remove debris, and serum-free DMEM was added. Scratch closure was observed and quantified at 0h, 12h, and 24h. ImageJ was used to analyze migration by measuring the initial scratch area (S_0_) and the healed scratch area (St). Migration percentage was calculated as: $\text{Migration }\left(\%\right)=\left(1-\text{St}/S_{0}\right)\times 100\%$ .

Transwell chamber assay: Cell suspensions were adjusted to 2 × 10^5^ cells/mL. Transwell chambers were pre-equilibrated in serum-free DMEM for 1 h. A total of 100 μL of the cell suspension was added to the upper chamber, while 500 μL of DMEM containing 10% FBS was introduced into the lower chamber. Chambers were incubated at 37°C with 5% CO_2_ for 24 h. After incubation, the Transwell chamber was removed, and cells on the top side of the chamber were gently removed using cotton swabs. Cells were fixed with 4% paraformaldehyde for 30 min at 37°C and then washed three times with PBS. Finally, the cells were stained with a 0.1% crystal violet solution. Afterward, images of five randomly selected fields of view were captured under an inverted microscope, and cell migration was quantified.

### *In vitro* BMSCs chondrogenic differentiation

BMSC suspensions were seeded into 6-well plates at a density of 2 × 10^5^ cells/mL and cultured under the previously described grouping conditions. Each group was supplemented with identical chondrogenic differentiation induction medium (composed of DMEM/F12 basal medium containing 50 μg/mL ascorbic acid, 100 nM dexamethasone, 1% ITS additive, 100 mg/mL sodium pyruvate, 50 μg/mL proline, and 10 ng/mL TGF-β3). After 7 days, the chondrogenic differentiation potential of the BMSCs was assessed via qRT-PCR, Western blot, and Alcian blue staining.

Total RNA was extracted from BMSCs using the RNAiso Plus Kit following the manufacturer’s protocols. The reference gene was glyceraldehydes 3-phosphate dehydrogenase (GAPDH). cDNA synthesis was performed using 1 μg of total RNA with the RevertAid First Strand cDNA Synthesis Kit. Quantitative real-time PCR (qRT-PCR) was conducted to analyze the relative expression levels of chondrogenic markers (Sox9, Acan, and Col2α1) using the 2^−ΔΔCT^ method. Primer sequences are detailed in supplementary Table S2. For protein-level validation, total cellular proteins were extracted and quantified via Western blot analysis. β-Actin served as an internal loading control for normalization.

The cells from each group after treatment were fixed with 4% paraformaldehyde (PFA), followed by Alcian blue staining for 30 min at 37°C. The cells were then observed by microscopy.

### Construction of rat knee joint cartilage defect model

6–8-week-old SD rats (n = 18) were numbered and randomly divided into three groups: Control group, CS/HCA hydrogel group (CS/HCA), KGN-loaded CS/HCA hydrogel group (CS-HCA@PLGA-PEG@KGN). Rats were anesthetized using pentobarbital sodium (0.2 g/mL), followed by shaving and sterilization of the knee area. A midline incision was made over the knee joint to expose the femoral trochlea. A full-thickness cartilage defect (2.0 mm in diameter, 1.5 mm in depth) was created in the center of the trochlea. After repeated saline irrigation, procedures varied according to group assignment: the cartilage defect in the control group was left untreated, and the incision was directly sutured. For the two experimental groups, the defects were filled with either the CS/HCA hydrogel or the CS-HCA@PLGA-PEG@KGN hydrogel. Postoperatively, rats were individually housed with unrestricted knee joint movement. The incision site was disinfected every 3 days.

### Chondrogenic induction *in vivo*

At 6 weeks postoperatively, the animals were euthanized by intravenous injection of an overdose of pentobarbital sodium, and the knee joints were harvested. First, a digital camera was used to capture images of the osteochondral defects. International Cartilage Repair Society (ICRS) scoring system was used to score the defect site (Van den Borne et al., 2007). Three specimens were randomly selected from each group for histological analysis. The specimens were soaked in 10% formalin overnight, decalcified, embedded in paraffin, and sectioned for staining. Hematoxylin and eosin (HE) and Collagen II immunohistochemical staining were performed for histological analyses. The sections were scored using a modified O’Driscoll histology scoring system (MODHS) (O'Driscoll et al., 2001).

### Statistical analysis

Statistical analysis was performed using IBM SPSS software 19.0 (IBM Corp., Armonk, NY, United States). Numerical data are expressed as mean ± standard deviation (M ± SD). One-way ANOVA was used for between-group comparisons, with statistical significance defined as P < 0.05.

## Results

### Characterization of PLGA-PEG@KGN nanoparticles

The synthesis route of PLGA-PEG@KGN is shown in Figure 1A, and the chemical structure of PLGA-PEG was confirmed by ^1^H NMR spectroscopy. As shown in Figure 1B, the PLGA spectrum exhibits a characteristic–CH3 peak at 1.47 ppm and a CH3CHOCH proton peak in the range of 5.19–5.21 ppm. After grafting PEG onto PLGA, a distinct–CH2CH2O- proton peak from PEG is clearly observed at 3.47–3.51 ppm, confirming the successful synthesis of PLGA-PEG.

As shown in Figures 1C–F, the fabricated PLGA-PEG nanoparticles are spherical with an average diameter of 157.2 nm and a surface Zeta potential of −1.7 mV. The PLGA-PEG@KGN nanoparticles exhibit an average diameter of 343.8 nm and a surface Zeta potential of −13.0 mV. The peak area obtained from the analysis of the sample using high-performance liquid chromatography (HPLC) was substituted into the standard curve, resulting in a calculated encapsulation efficiency of 88.86% for the nanoparticles loaded with KGN (Figures 1G,H). UV-Vis spectrophotometry analysis (Figures 1I,J) demonstrated an encapsulation efficiency of 73.2%, which is relatively close to the result obtained by HPLC. Additionally, the drug loading capacity of KGN was calculated to be 6.82% based on UV-Vis spectrophotometry analysis.

### Characterization of CS-HCA dynamic hydrogel

The synthesis route of CS-HCA polymer is shown in Figure 2A. UV spectroscopy analysis revealed that CS exhibited no significant absorption peaks in the range of 200–500 nm. However, after HCA grafting, distinct characteristic peaks of HCA appeared at 226 and 281 nm (Figure 2B), corresponding to the phenolic structures in HCA, thereby confirming the successful grafting of CS-HCA.

To verify structural integrity, FT-IR analysis was conducted on CS, HCA, and CS-HCA (Figure 2C). The CS spectrum originally lacking benzene ring vibration peaks exhibited distinct benzene skeleton vibrations and C=C harmonic peaks after HCA grafting, consistent with UV findings. ^1^H-NMR analysis (Figure 2D) identified CS methyl group (CH3-) at 1.94 ppm, methylene groups (-CH2-) at 3.34 ppm and -CH2O- at 2.62 ppm. CS aromatic protons (H-1–6) appeared at 4.7, 2.91, and 3.57–3.81 ppm, while HCA aromatic protons (H-7–9) showed multiplets at 6.52–6.74 ppm. These spectral analyses collectively confirmed the successful preparation of CS-HCA. The grafting rate of CS-HCA was calculated to be 16.67% based on the ratio of the integral area of HCA characteristic peak in ^1^H-NMR to that of H_2_ peak in CS, and the actual yield was 58.33%.

As demonstrated in Figures 2E,G, the CS-HCA hydrogel exhibits injectability and self-healing properties. The hydrogel solution could be injected via a syringe to form hydrogels of arbitrary shapes, enabling seamless filling of irregular cartilage defects. When cut into halves, the hydrogel exhibited self-healing capability upon contact without visible cracks. Adhesion tests demonstrated that the hydrogel adhered firmly to plastic, glass, and skin surfaces (Figure 2F). In porcine skin adhesion assays (Figure 2H), the hydrogel exhibited strong bioadhesion, maintaining structural integrity under 80% tensile deformation with an adhesion strength of 1,150 kPa.

To further verify the self-healing properties of the hydrogel, we conducted dynamic mechanical analysis. As demonstrated in Figures 2I,J, the compressive modulus of the normally synthesized hydrogel reaches ∼195 kPa, while that of the self-healed hydrogel after fracture shows a slight decrease but still achieves ∼170 kPa, indicating its retained strong compressive performance. In terms of tensile mechanical properties, the tensile modulus of the intact hydrogel reaches ∼75 kPa, whereas the self-healed hydrogel maintains a modulus of ∼70 kPa. These results demonstrate that the CS-HCA hydrogel possesses self-healing capability, and the healed hydrogel can still maintain robust compressive and tensile properties.

To analyze the dynamic mechanical properties of the CS-HCA hydrogel, we conducted rheological analysis. The frequency sweep test results demonstrated that within the linear viscoelastic region, the storage modulus (G′) of both the CS-HCA hydrogel and the CS-HCA@PLGA-PEG@KGN hydrogel was consistently greater than the loss modulus (G″), indicating a gel-like state. As shown in Figures 2K,L, the rheological properties of the CS-HCA hydrogel exhibited no significant changes before and after loading KGN nanoparticles. The small difference between G′ and G″ for both the CS-HCA hydrogel and the CS-HCA@PLGA-PEG@KGN hydrogel suggests a certain degree of fluidity, making them suitable for injectable therapies.

### Microstructure of CS-HCA hydrogel

**FIGURE 3:**
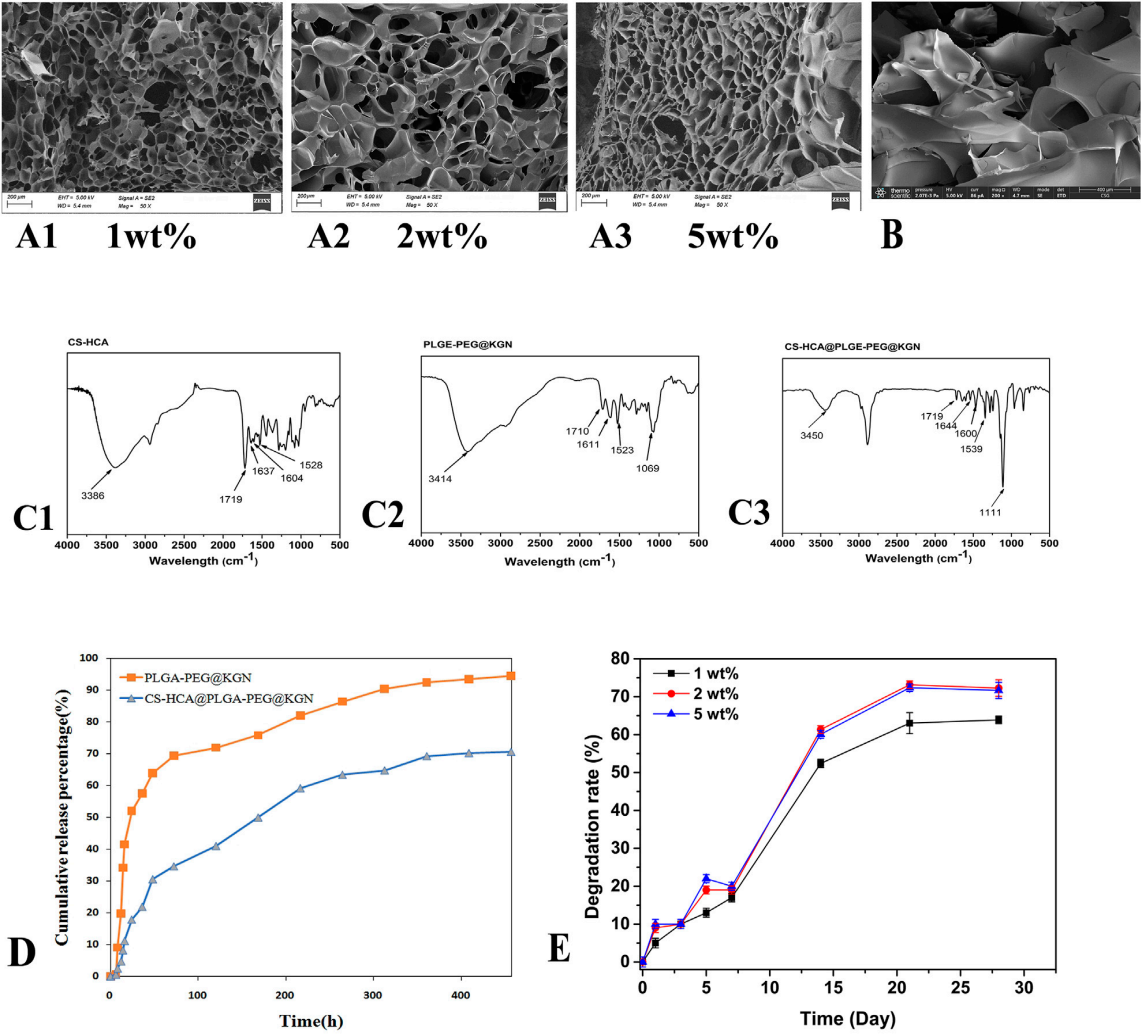
**(A1–A3)** SEM images of CS-HCA hydrogels with concentrations of 1wt%, 2 wt%, and 5wt%. **(B)** SEM image of CS-HCA@PLGA-PEG@KGN hydrogel. **(C)** FT-IR spectra of CS-HCA hydrogel **(C1)** PLGA-PEG@KGN nanoparticles **(C2)** and CS-HCA@PLGA-PEG@KGN hydrogel **(C3) (D)** Drug release profiles of PLGA-PEG@KGN nanoparticles and CS-HCA@PLGA-PEG@KGN hydrogels. **(E)** Degradation profiles of CS-HCA hydrogels at different concentrations.

As shown in Figure 3A, hydrogel samples formed at different concentrations exhibited typical three-dimensional microporous structures, with porosities ranging between 69% and 80%. The internal structure displayed a heterogeneous but continuous pore network with smooth surfaces, where pore diameters primarily ranged from 20 to 200 μm. Notably, the 2 wt% hydrogel exhibited slightly larger pore sizes compared to the other two groups, whereas the 5 wt% hydrogel demonstrated the highest porosity of approximately 79%. Nanoparticles were successfully attached to the surfaces of the internal pores (Figure 3B). This interconnected pore structure facilitates cell adhesion, nutrient diffusion, and metabolic waste exchange.

### Drug release and degradation properties of CS-HCA@PLGA-PEG@KGN dynamic hydrogel

We first investigated the interaction between CS-HCA hydrogels and PLGA-PEG@KGN nanoparticles through FT-IR analysis. As shown in Figure 3C, the FT-IR spectrum of CS-HCA hydrogels displayed characteristic peaks of -OH and -NH_2_ at approximately 3,386 cm^-1^. The characteristic peaks of C=O, -CONH-, and aromatic rings were observed at 1719, 1,637, 1,604, and 1,528 cm^-1^. In the FT-IR spectrum of PLGA-PEG@KGN nanoparticles, the -OH characteristic peak appeared at around 3,414 cm^-1^, while the C-O-C peak was detected at 1,069 cm^-1^. The C=O peak was observed at 1710 cm^-1^, and the characteristic peaks of the benzene ring from KGN were found at 1,611 and 1,523 cm^-1^. The FT-IR spectrum of CS-HCA@PLGA-PEG@KGN hydrogels revealed that the -OH and -NH_2_ characteristic peaks of CS-HCA and PLGA-PEG@KGN shifted to 3,386 cm^-1^, while the C=O peak shifted to 1,111 cm^-1^. The benzene ring and -CONH- characteristic peaks of CS-HCA shifted to 1,539, 1,644, and 1,600 cm^-1^. These results suggest that CS-HCA hydrogels may achieve effective loading onto PLGA-PEG@KGN nanoparticles through electrostatic or van der Waals interactions.

UV-Vis spectrophotometry was employed to evaluate the drug release kinetics of PLGA-PEG@KGN nanoparticles and CS-HCA@PLGA-PEG@KGN hydrogels. The results (Figure 3D) demonstrated that both systems exhibited sustained drug release for over 3 weeks. Approximately 75% of KGN was released from the nanoparticles within the first week, compared to only 50% from the CS-HCA@PLGA-PEG@KGN hydrogel. By the third week, cumulative release reached 94% for the nanoparticles and 70% for the hydrogel. The prolonged drug release from the hydrogel, compared to the nanoparticles alone, indicates that the hydrogel achieves dual-release functionality through effective adsorption of nanoparticles.

The degradation profiles in Figure 3E revealed that CS-HCA hydrogels at different concentrations degraded by approximately 20% in the first week. The degradation rate accelerated in the second week, reaching 50%–60%, before slowing again to stabilize between 60% and 75% by the fourth week. This degradation pattern allowed the hydrogel to initially provide temporary mechanical support for cell adhesion and proliferation, followed by gradual degradation during cartilage regeneration to facilitate the deposition of newly formed tissue.

### Effects of CS-HCA@PLGA-PEG@KGN hydrogel on BMSCs proliferation, migration, and differentiation

**FIGURE 4:**
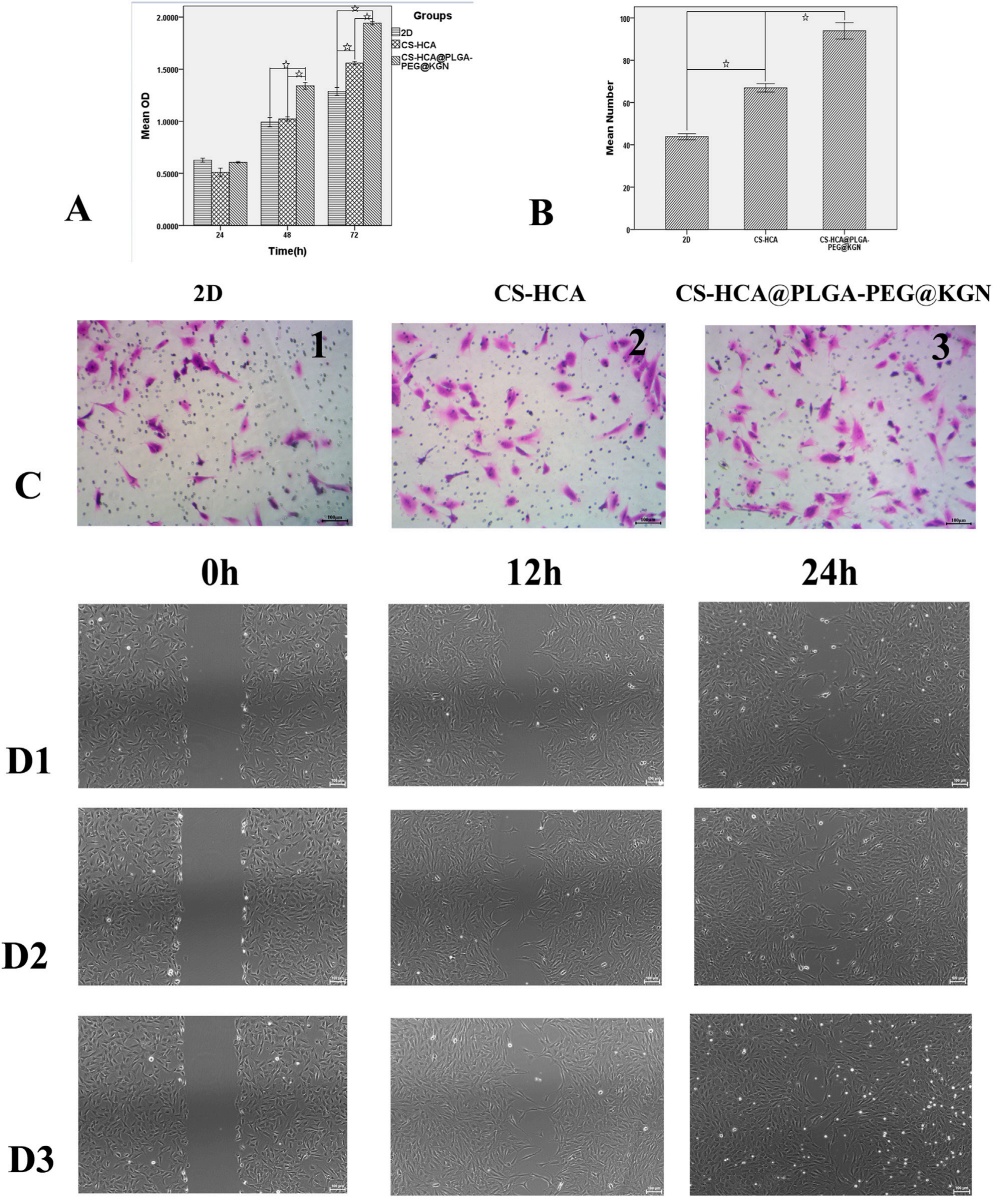
*In vitro* effects of CS-HCA@PLGA-PEG@KGN hydrogels on the proliferation and migration of BMSCs. **(A)** Assessment of BMSCs proliferation across three groups using CCK-8 assay. **(B)** Quantitative comparison of migrated BMSCs in three groups at 24-h post-treatment. **(C)** Assessment of BMSCs migration across three groups using Transwell chamber assay. **(D)** Assessment of BMSCs migration across three groups using Scratch wound healing assay **(D1)** 2D group, **(D2)** CS-HCA hydrogel group, **(D3)** CS-HCA@PLGA-PEG@KGN hydrogel group). (☆P < 0.05)

*In vitro* experiments demonstrated that the CS-HCA@PLGA-PEG@KGN hydrogel significantly enhanced the proliferation, migration, and chondrogenic differentiation of BMSCs. As shown in Figure 4A, the CCK-8 assay revealed that BMSCs cultured on the CS-HCA@PLGA-PEG@KGN hydrogel exhibited a markedly higher proliferation rate compared to those in the 2D group and the CS-HCA group (p < 0.05). Scratch assays and Transwell migration assays (Figures 4B–D) further indicated that cell migration rates in the CS-HCA@PLGA-PEG@KGN group were superior to those in the other two groups, while the CS-HCA group outperformed the traditional 2D culture group (p < 0.05).

**FIGURE 5:**
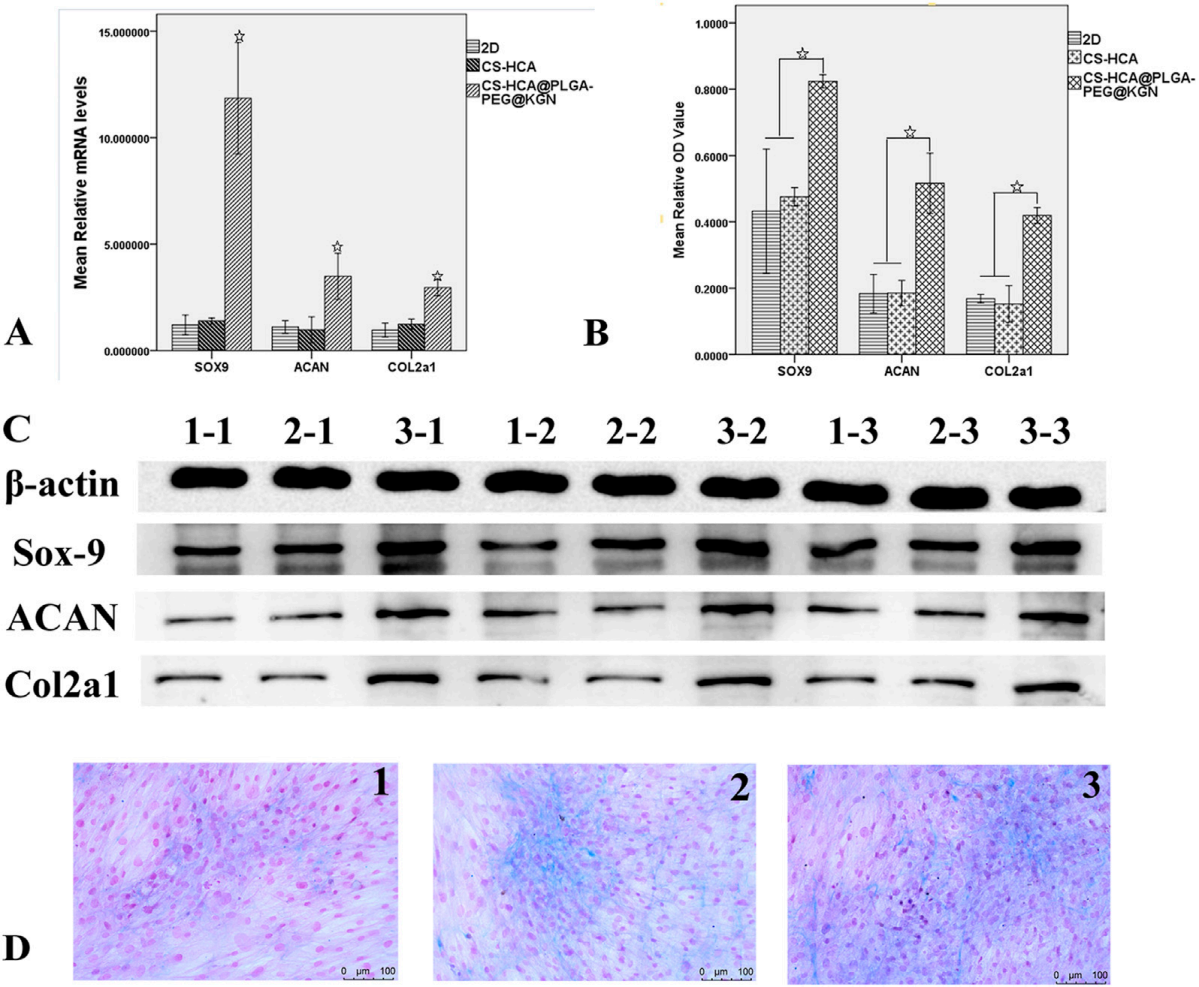
*In vitro* effects of CS-HCA@PLGA-PEG@KGN hydrogels on the chondrogenic differentiation of BMSCs. **(A)** qRT-PCR quantification of Sox9, Acan, and Col2α1 mRNA expression levels of BMSCs in 2D, CS-HCA,and CS-HCA@PLGA-PEG@KGN groups. **(B)** Quantitative comparison of Sox9, Acan and Col2α1 protein expression levels of BMSCs in three groups. **(C)** Western blotting analysis of Sox9, Acan, and Col2α1 protein expression levels of BMSCs in 2D (1–1,1–2,1–3), CS-HCA (2–1,2–2,2–3) and CS-HCA@PLGA-PEG@KGN (3–1,3–2,3–3) groups. **(D)** Alcian blue staining of sulfated glycosaminoglycan (GAG) content in 2D **(D1)** CS-HCA **(D2)** and CS-HCA@PLGA-PEG@KGN **(D3)** groups. (☆P < 0.05)

qRT-PCR and Western blot analyses (Figure 5) demonstrated that the mRNA and protein expression levels of chondrogenic markers (Sox9, Aggrecan, and COL2A1) in the CS-HCA@PLGA-PEG@KGN group were significantly upregulated compared to the other groups (p < 0.05). Alizarin Blue staining showed noticeable blue staining across all three groups after chondrogenic induction, indicating glycosaminoglycan synthesis. Notably, the CS-HCA@PLGA-PEG@KGN hydrogel group exhibited the most intense blue staining, with distinct cell aggregates, suggesting the highest glycosaminoglycan content.

These findings confirm that the CS-HCA@PLGA-PEG@KGN hydrogel provides a favorable three-dimensional microenvironment for stem cell growth and differentiation. Its porous structure and sustained KGN release promote cell adhesion on and within the scaffold, thereby enhancing BMSC proliferation, migration, and chondrogenic differentiation capacity.

### Gross morphological evaluation of cartilage defect repair

**FIGURE 6:**
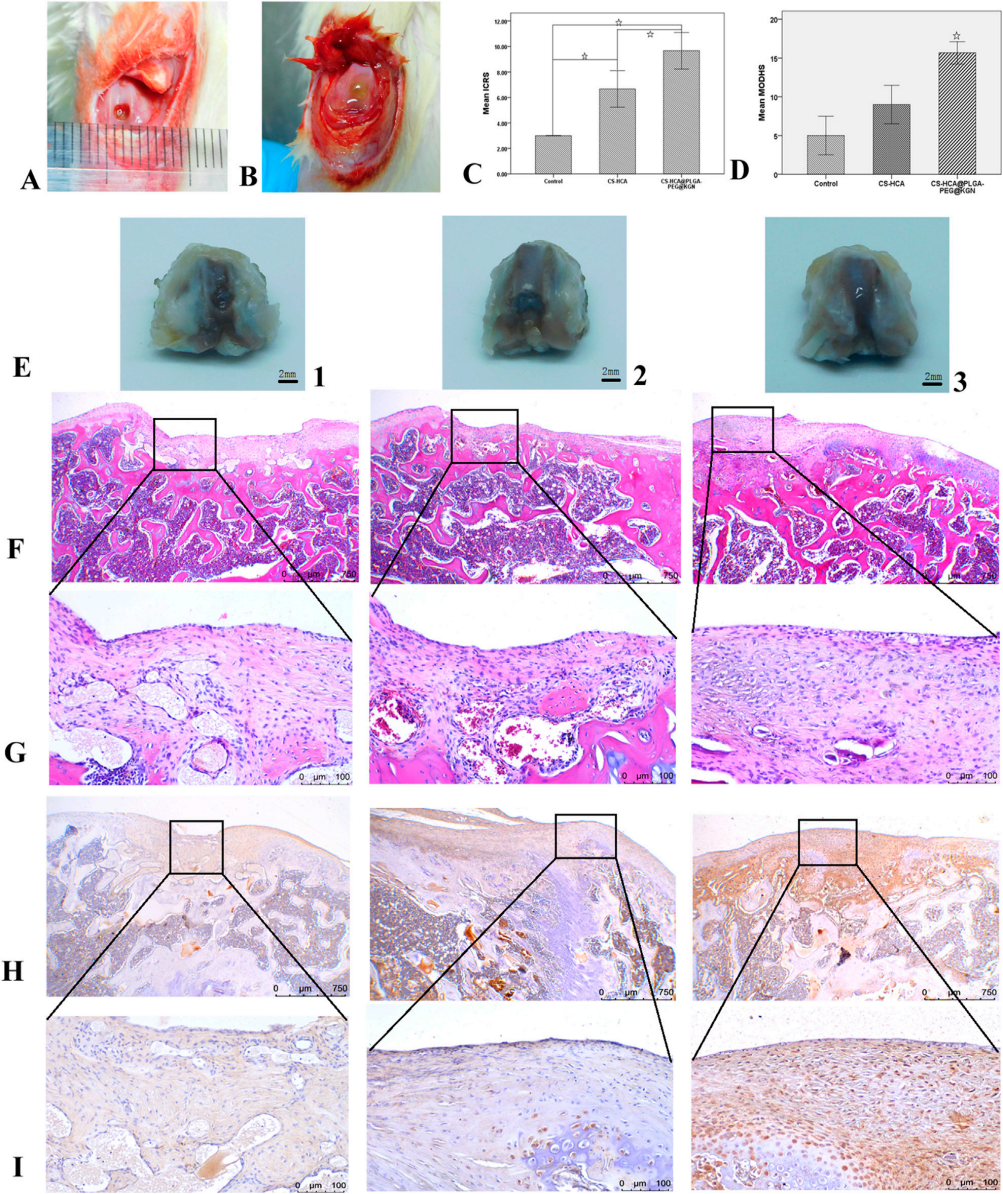
Healing efficacy of *in vivo* cartilage defects. **(A,B)** Full-thickness cartilage defect model of rat femoral trochlea **(A)** and hydrogel implantation in osteochondral defects **(B)**. **(C)** ICRS scores of neocartilage in three groups. **(D)** The modified O’Driscoll histological evaluation of neocartilage in three groups. **(E)** Gross morphological observation of neotissue in Control (1), CS-HCA (2) and CS-HCA@PLGA-PEG@KGN (3) groups. **(F,G)** HE staining of neocartilage at ×40 (F) and ×200 **(G)** magnification in three groups. **(H,I)** Col II immunohistochemical staining at ×40(H) and ×200(I) magnification in three groups. (☆P < 0.05).

Figures 6A-C illustrate the surgical procedure for establishing the rat cartilage defect model and the ICRS scoring results. At 6 weeks post-surgery, gross morphological evaluation revealed distinct repair outcomes across the three groups in the cartilage defect regions (Figure 6E). While all treatments demonstrated new tissue growth at the defect sites, the CS-HCA@PLGA-PEG@KGN hydrogel group exhibited the most optimal repair outcome. The control group (Figure 6E1) displayed incomplete defect filling with localized depressions, and a clear boundary between the newly formed tissue and surrounding healthy cartilage. The CS-HCA hydrogel group (Figure 6E2) achieved approximately 80% defect coverage, though the newly formed tissue remained slightly depressed compared to the intact cartilage. In contrast, the CS-HCA@PLGA-PEG@KGN group (Figure 6E3) showed complete filling of the defect with translucent and smooth tissue, closely resembling the appearance of adjacent normal cartilage. The repaired area was well-integrated without visible fissures, inflammatory infiltration, or residual gel components. ICRS scoring (Figure 6C) confirmed that the CS-HCA@PLGA-PEG@KGN group attained the highest score, indicating superior cartilage repair efficacy.

### Histological and immunohistochemical analysis of cartilage defect repair

Tissue samples were sectioned and subjected to histological staining analyses to further evaluate cartilage regeneration. HE staining (Figures 6F,G) revealed that the control group exhibited fibrous tissue filling the defect site, with a clear boundary and noticeable depression compared to surrounding cartilage. Type II collagen staining (Figures 6H,I) demonstrated weaker staining intensity in the repaired tissue compared to adjacent normal cartilage. The CS-HCA hydrogel group showed incomplete defect repair, though with reduced depression depth relative to the control group. While the repaired tissue was closely integrated with surrounding cartilage, chondrocyte density was lower, and type II collagen staining intensity remained inferior to that of normal cartilage. In contrast, the CS-HCA@PLGA-PEG@KGN hydrogel group achieved complete filling of the defect with transparent cartilage-like tissue, flush with the surrounding normal cartilage and exhibiting seamless interface integration. Although chondrocyte arrangement within the repaired area partially deviated from natural cartilage morphology, type II collagen staining was more uniform and robust compared to the other groups. Modified O’Driscoll histology scoring (Figure 6D) confirmed that the CS-HCA@PLGA-PEG@KGN hydrogel group attained the highest score, indicating optimal cartilage regeneration outcomes.

## Discussion

The restoration of articular cartilage defects is still a great clinical challenge because of the limited intrinsic potential for self-healing (Xiang et al., 2022; Hu et al., 2021). Bone marrow stimulation techniques include drilling and the microfracture technique, which aim to recruit BMSCs to repair cartilage defects. In young patients with cartilage defects, the microfracture technique has become a first-line clinical treatment due to its cost-effectiveness, minimally invasive nature, and procedural simplicity. However, due to insufficient recruitment of BMSCs or the impact of the local inflammatory microenvironment after cartilage injury on stem cell differentiation into chondrocytes, the newly formed tissue is predominantly fibrocartilage with poor integration into the surrounding cartilage. Fibrocartilage gradually degenerates over time, resulting in poor long-term outcomes (Mithoefer et al., 2009). Microfracture combined with tissue engineering technology can improve the microenvironment around damaged cartilage, promote stem cell migration and differentiation into chondrocytes, thereby enhancing the repair effect (Frehner and Benthien, 2018).

In cartilage engineering scaffold materials, injectable hydrogels exhibit unique advantages and significant clinical potential due to their ability to repair complex-shaped defects and enable minimally invasive implantation. The chitosan backbone, rich in amino groups, is an ideal material for constructing dynamic hydrogels based on imine bonds. In this study, a bioadhesive dynamic hydrogel was successfully prepared by crosslinking CS-HCA with aldehyde-terminated four-armed polyethylene glycol (AF-PEG). AF-PEG contains abundant aldehyde groups and can rapidly form a gel within 2 min when mixed with CS-HCA under physiological conditions. This hydrogel demonstrates excellent injectability and self-healing properties, meeting the requirements for clinical minimally invasive implantation. Compared to other aldehyde-based crosslinkers commonly used in chitosan hydrogels, the CS-HCA hydrogel synthesized in this study exhibits superior toughness, with a compressive strength of up to 195 kPa. Moreover, the fractured and self-healed hydrogel retains mechanical properties similar to those of the original hydrogel, achieving a compressive modulus of 170 kPa and a tensile modulus of 70 kPa. Research indicates that the phenolic hydroxyl groups in catechol can also promote hydrogel self-healing through non-covalent interactions such as hydrogen bonding and π-π stacking (Li et al., 2019). The CS-HCA hydrogel contains abundant dynamic imine bonds and catechol groups. These reversible interactions can break during hydrogel deformation, effectively dissipating energy and thereby enhancing the hydrogel’s toughness, enabling it to withstand repeated loads and mimic the biomechanical properties of natural cartilage.

In addition to its injectability and self-healing properties, the CS-HCA@PLGA-PEG@KGN hydrogel exhibits robust bioadhesive capabilities. CS-HCA polymer contains a high density of free phenolic hydroxyl groups, which can form covalent bonds with amino and thiol groups in organic tissues, as well as establish non-covalent interactions such as π-π stacking with various inorganic surfaces, thereby endowing the CS-HCA hydrogel with superior bioadhesive capabilities (Zhou et al., 2022). As illustrated in Figure 2, the hydrogel demonstrates strong adhesion to both organic and inorganic substrates. Even under a tensile force of up to 1,150 kPa, the hydrogel-bonded porcine skin samples remained intact, highlighting its exceptional bioadhesive strength. The catechol groups further mediate interfacial adhesion in wet environments, with studies confirming their superior underwater adhesive performance (Guo et al., 2020). Following implantation into cartilage defects, the CS-HCA@PLGA-PEG@KGN hydrogel forms stable adhesion with surrounding host tissues, ensuring secure retention without displacement from the implantation site. Researches indicate that the catechol groups exhibit high protein affinity, enabling them to anchor endogenous growth factors from bodily fluids and blood, while also capturing signaling proteins secreted by adherent cells on the hydrogel surface (Zhao et al., 2024). These retained growth factors and signaling proteins synergistically enhance cellular adhesion, thereby establishing a microenvironment conducive to tissue regeneration. Its injectability, self-healing capacity, and strong bioadhesion collectively fulfill the requirements for minimally invasive arthroscopic procedures, underscoring its significant translational potential in clinical applications. *In vitro* degradation analysis revealed that the hydrogel exhibited minimal mass loss during the initial 7 days, followed by a progressive degradation profile, with approximately 30%–40% of the initial mass retained at day 28. This controlled degradation kinetics not only promoted early-stage cell adhesion and proliferation but also provided structural support for neocartilage deposition in subsequent phases.

Studies demonstrate that the three-dimensional porous architecture of chitosan-based hydrogels mimics the topological structure of natural cartilage extracellular matrix (ECM). The CS-HCA@PLGA-PEG@KGN hydrogel features a characteristic 3D porous structure with a porosity of 69%–80% and pore sizes precisely controlled within the range of 20–200 μm. Research indicates that macropores (150–200 μm) facilitate cell infiltration, while micropores (20–50 μm) increase the surface area-to-volume ratio for efficient ECM protein adsorption (Lyu et al., 2022). Kim et al. reported that a macroporous polyvinyl alcohol (PVA) scaffold facilitated chondrocyte migration from host cartilage into scaffold and improved interface integration in an *in vitro* cartilage defect model (Kim et al., 2017). The microporous structure also contributes to sustained drug release. Through FT-IR analysis of CS-HCA hydrogels before and after loading PLGA-PEG@KGN nanoparticles, our results indicated that the nanoparticles could be adsorbed into the hydrogel via hydrogen bonds or van der Waals forces. Drug release profiling revealed that PLGA-PEG@KGN nanoparticles released approximately 75% of their KGN payload within 1 week, whereas the CS-HCA@PLGA-PEG@KGN hydrogel released only 50% during the same period. By week 3, cumulative release reached 94.5% for nanoparticles compared to 70.6% for the hydrogel. These results confirm the hydrogel’s capacity to effectively retain nanoparticles and establish a dual sustained-release mechanism for KGN delivery.

It is well-established that KGN promotes the differentiation of BMSCs into chondrocytes. Studies have demonstrated that KGN selectively activates the BMP/Smad1/5 pathway while inhibiting the Wnt/β-catenin pathway, thereby upregulating the expression of SOX9 and COL2A1 and downregulating COL10A1 to prevent chondrocyte hypertrophy (Cai et al., 2019). Researchers have utilized KGN combined with a self-made double-network hydrogel to repair rabbit knee articular cartilage defects, achieving favorable outcomes (Chen et al., 2022). RNA sequencing and Gene Ontology (GO) analysis revealed significant upregulation of genes involved in cell proliferation, chondrogenic differentiation, and cartilage matrix synthesis within the regenerated tissue, accompanied by downregulation of proteolytic and inflammatory response-related genes. Notably, KEGG pathway enrichment analysis demonstrated enhanced activation of metabolic pathways linked to extracellular matrix (ECM) production—including focal adhesion and ECM-receptor interaction—while pathways associated with matrix degradation and inflammatory signaling were markedly suppressed. In our study, the CS-HCA@PLGA-PEG@KGN hydrogel demonstrated excellent biocompatibility, enhancing BMSC proliferation, migration, and chondrogenesis. The abundant 3D microporous structure of the hydrogel, combined with its sustained-release KGN, provides a favorable microenvironment for BMSC survival and chondrogenic differentiation.

BM-MSCs are abundant yet prone to chondrocyte hypertrophy and osteogenic differentiation (Zhang et al., 2020). In injured or degenerative joints, the local inflammatory microenvironment compromises cell survival and differentiation, severely impairing repair outcomes (Roseti et al., 2019). Studies demonstrate that KGN enhances stem cell chondrogenesis and inhibits cartilage degradation in IL-1β-induced inflammatory joints (Fan et al., 2020). KGN’s enzymatic metabolite 4-ABP (4-aminobiphenyl) promotes MSCs proliferation and chondrogenesis via the PI3K-Akt pathway, repairing osteoarthritis cartilage damage (Zhang et al., 2019). Additionally, KGN alleviates joint inflammation and prevents degeneration through the miR-146a/NRF2 axis (Hou et al., 2021). In our study, animal experiments revealed the CS-HCA@PLGA-PEG@KGN hydrogel group achieved complete cartilage regeneration in defect zones at 6 weeks post-surgery, with seamless integration into surrounding tissue. Although chondrocyte arrangement within the repaired area partially deviated from natural cartilage morphology, type II collagen staining was more uniform and robust compared to the other groups.

However, this study has several limitations. First, the observation time points in animal experiments were limited, and the mechanistic studies on cartilage regeneration remain superficial. Second, the hydrogel still struggles to replicate the complex hierarchical architecture of natural cartilage, resulting in structural discrepancies between the newly formed cartilage and normal tissue. To address these challenges, future studies could integrate 3D bioprinting technology with the spatiotemporal synergy of multiple bioactive factors (e.g., TGF-β, FGF) to engineer biomimetic hydrogels that recapitulate the structural complexity of articular cartilage, thereby promoting hierarchical cartilage regeneration.

## Conclusion

In this study, we successfully developed a CS-HCA@PLGA-PEG@KGN bioadhesive dynamic hydrogel based on the design principles of bioadhesion, dynamic imine bonds, and sustained release of KGN bioactive factors. *In vitro* experiments demonstrated that the hydrogel exhibits excellent bioadhesive properties, injectability, and self-healing capabilities. Its microporous structure and sustained release of KGN effectively promoted the proliferation, migration, and chondrogenic differentiation of BMSCs. Animal studies revealed that the hydrogel significantly enhanced the repair of cartilage defects in rat knee joints, with newly formed cartilage resembling hyaline-like tissue and demonstrating seamless integration with surrounding native cartilage. Furthermore, the hydrogel displayed rapid gelation under physiological conditions, adaptability to irregular defect geometries, and operational simplicity with a controllable fabrication process, highlighting its promising potential for clinical applications in cartilage regeneration.

## Data Availability

The datasets presented in this study can be found in online repositories. The names of the repository/repositories and accession number(s) can be found in the article/Supplementary Material.
