# Supplementary material for: Bioadhesive chitosan hydrogel with dynamic covalent bonds and sustained kartogenin release for endogenous cartilage regeneration
Source: Front Bioeng Biotechnol. 2025 Jul 29;13:1606726. doi: 10.3389/fbioe.2025.1606726 (PMC12339519; doi:10.3389/fbioe.2025.1606726)
Supplement: Supplementary file 1 [file Table1.docx]

Table S1. Reagents and Instruments

| **Reagents and Instruments** | **Manufacturer** |
| --- | --- |
| ^1^H NMR spectroscopy | JEOL, Japan |
| Dynamic Light Scattering (DLS) | Brookhaven Instruments, United States |
| Electrophoretic Light Scattering (ELS) | Brookhaven Instruments, United States |
| Transmission Electron Microscopy (TEM) | Hitachi, Japan |
| UV-Vis Spectrophotometry | Shimadzu, Japan |
| FT-IR Spectroscopy | Thermo Scientific, United States |
| Scanning Electron Microscopy (SEM) | Hitachi, Japan |
| DMEM/F12 Culture medium | Gibco, United States |
| Fetal bovine serum (FBS) | Gibco, United States |
| CCK-8 Assay Kit; | Beyotime Biotechnology, China |
| TGF-β3 | Peprotech, United States |
| Alcian Blue Staining Kit | Solarbio, China |
| RNAiso plus | Takara, Japan |
| Hifair® III 1st Strand cDNA Synthesis Kit(gDNA digester plus) | YEASEN, China |
| Hieff UNICON® Universal Blue Qpcr SYBR Green Master Mix | YEASEN, China |
| Primary antibodies:  SOX9 (rabbit-derived),  Aggrecan (rabbit-derived),  Col2a1 (rabbit-derived),  Collagen Type II (COL II) (rabbit-derived) | Abcam ,United Kingdom |
| Secondary antibody: HRP-conjugated Goat Anti-Rabbit IgG | Abcam ,United Kingdom |
| Paraformaldehyde | Shanghai Bioengineering, China |
| Multifunctional Microplate Reader | Thermo Fisher, United States |
| Transwell Chamber | Corning, United States |
| StepOne Plus Real-Time PCR System | ABI, United States |
| Electrophoresis Apparatus | Bio-rad, United States |
| Gel Imaging System with Integrated Voltage Stabilizer for DNA Electrophoresis | Tanon, China |
| Tanon-4200 Gel Imaging System | Tanon, China |
