# Supplementary material for: Bioadhesive chitosan hydrogel with dynamic covalent bonds and sustained kartogenin release for endogenous cartilage regeneration
Source: Front Bioeng Biotechnol. 2025 Jul 29;13:1606726. doi: 10.3389/fbioe.2025.1606726 (PMC12339519; doi:10.3389/fbioe.2025.1606726)
Supplement: Supplementary file 2 [file Table2.docx]

Table S2. Oligonucleotide primer sequences utilized for q RT-PCR

| Gene | Premier 5’-3’ |
| --- | --- |
| GADPH | F: AGATCCCTCCAAAATCAAGTGG |
|  | R: GGCAGAGATGATGACCCTTTT |
| COL2A1 | F: GGATGTATGGAAGCCCTCGTC |
|  | R: GTTCTCCTTTCTGCCCCTTTGG |
| SOX-9 | F: AACCCCTCCTACCCAACCAT |
|  | R: TGGTGAGCTGTGTGTAGACG |
| Aggrecan | F: CCTGTGTGAGATCGACCAGGAG |
|  | R: CCATCAGACCAGCGGAAGTC |
